# A Novel Splice-Site Variation in *COL5A1* Causes Keratoconus in an Indian Family

**DOI:** 10.1155/2019/2851380

**Published:** 2019-10-20

**Authors:** Qinghong Lin, Lin Zheng, Zhengwei Shen, Liming Jie

**Affiliations:** Affiliated Xiamen Eye Center, Eye Institute of Xiamen University, Xiamen 361000, China

## Abstract

**Objective:**

This study aims to clarify the association between keratoconus (KC) and potential pathogenic genetic variants in a three-generation South Indian family.

**Methods:**

In the present study, a three-generation KC family, which comprised 10 affected patients and nine unaffected individuals, was recruited. The family history and necessary ophthalmological exams, such as visual acuity and slit-lamp, were performed for all participants. Genomic DNA was extracted from peripheral blood leukocytes, and whole exome sequencing (WES) was performed using the genomic DNA of the proband (III:4) and two other family members (III:2, III:3). The acceptor-splice-site mutation was validated and verified using polymerase chain reaction (PCR) and Sanger sequencing. Gene functions and pathways associated with the identified mutations were subjected to *in silico* analysis.

**Results:**

A novel *COL5A1* acceptor-splice-site mutation IVS50-4C > G was found in the 10 affected individuals in the three-generation KC family, but this was not found in any of the unaffected family members or unrelated healthy individuals. Gene functional analysis using the SpliceMan and ExonScan software predicted that the splice-site mutation was potentially associated with KC pathogenesis. This mutation might affect the assembly of the collagen triple helix.

**Conclusion:**

The present study confirmed the association between the *COL5A1* gene and KC and identified a novel *COL5A1* acceptor-splice-site mutation (IVS50-4C > G) in intron 50, which may affect the splicing of the adjacent exon 50.

## 1. Introduction

Keratoconus (KC, OMIM 14830) is the irreversible progressive degeneration of the cornea that can distort vision. KC is characterized by the thinning of the central cornea and change in corneal shape, thereby leading to myopia and irregular astigmatism. Although the majority of KC cases are sporadic, genes correlated with KC, including *VSX1*, have been reported [1, 2]. Furthermore, it has been reported that approximately 6–10% of KC patients have a familial history of the disease [3, 4]. Both dominant and recessive inherent KC are often associated with variable phenotypes and incomplete penetrance [5, 6]. Although many gene variants at multiple chromosomal loci have been identified to be associated with KC to date, the inconsistencies in the roles of these variants among studies warrant further confirmation [7, 8]. In the past few years, genome-wide association analyses have revealed that the heritable trait—central corneal thickness (CCT)—has been found to be associated with KC [9].

Collagen, which determines the shape and strength of the cornea, is the primary component of the corneal structure. The arrangement of collagen fibrils, which are the main constituents of the corneal stroma, determines the biomechanical properties of the cornea, such as corneal curvature, transparency, CCT, and intraocular pressure [10]. Various collagen genes, such as *COL1A1*, *COL1A2*, and *COL5A1*, have been reported to be strongly associated with the changes in CCT [11–13]. In addition, the inactivation of the *COL8A1 and COL8A2* genes has reportedly led to the thinning of the central cornea [14]. The uneven distribution and abnormal orientation of the collagen fibrils were observed in the KC cornea, and this has been reported to be associated with CCT and the changes in corneal curvature [15].

Genetic determinants, including the *COL5A1* gene, have been identified to be associated with CCT [4, 12]. Notably, a KC-susceptibility locus at 9q34, which comprises single nucleotide polymorphisms (SNPs) such as rs1536482, has been reported to regulate CCT in the KC cornea [16]. To date, there are few reports on SNPs associated with both CCT and KC, either within or near the collagen genomic locus. The present study is the first to identify a novel acceptor-splice-site mutation in *COL5A1* from 10 moderate-to-severe KC members of a three-generation Indian family.

## 2. Materials and Methods

### 2.1. Ethics Statement

All experiments that involved human subjects were performed with the approval of the Institutional Review Board of Xiamen University (Xiamen, China), and these experiments were conducted according to the principles in the Declaration of Helsinki.

### 2.2. Participants and Procedures

**Figure 1:**
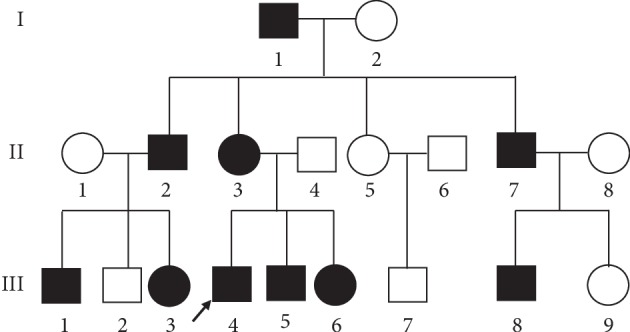
The genogram of the Indian family with kerotoconus. The squares and circles represent males and females, respectively. The solid symbols indicate the patients, while the open symbols indicate the unaffected individuals. The proband is denoted by the arrow.

In the present study, a three-generation South Indian family with KC, which included 10 affected members and nine unaffected relatives (Figure 1), was recruited from the Xiamen Eye Center, China. In addition, 120 healthy unrelated South Indian individuals were enrolled into the present study during the routine medical fitness examination at Xiamen University, China. All participants in the present study underwent comprehensive ophthalmological examinations, including visual acuity, slit-lamp, and imaging with a Scheimpflug camera system (Pentacam, Oculus Optikgerate GmbH, Wetzlar, Germany).

### 2.3. Whole Exome Sequencing (WES) and Data Analysis

Written informed consent was obtained from each participant included in the present study. Venous blood samples (5 mL) were collected in EDTA vacutainers (BD, San Jose, CA, USA), and the genome DNA was extracted from the peripheral blood leukocytes of KC patients and healthy individuals using a QIAamp DNA Blood Mini Kit (QIAGEN Science, Germantown, MD, Netherlands).

The WES of the genomic DNA of the proband (III:4) and two other members in the family (III:2 and III:3) was performed using the HiSeq2000 (Illumina, USA) with 101 base pairs paired-end reads. The enrichment of exonic sequences was achieved using a SureSelectXT Human All Exon V.2 Kit (50 Mb; Agilent Technologies, Inc., Santa Clara, CA, USA). Exome sequencing data processing, base calling, and primary data analysis were performed using the Illumina Real-Time Analysis (RTA) version 1.12.4 and Illumina's CASAVA pipeline 1.8.2 with default parameters. The paired-end reads were aligned to the reference human genome (hg19/GRCh37) using the Burrows–Wheeler Aligner (BWA v0.7.17) [17]. KC-associated and/or CCT-associated variants with a minor allele frequency of ≤1% in the 1000 Genomes, ExAC, and HapMap populations were extracted from the exome sequencing data. Potentially pathogenic variants that were only shared by patients III:3 and III:4 within the 19-member family, but were not observed in healthy individual III:2, were considered as preliminary candidate variants.

### 2.4. Variant Validation

Potential KC-related pathogenic variants identified from the WES were confirmed by comparing the results with those of the unaffected family members and all unrelated healthy individuals using polymerase chain reaction (PCR) and Sanger sequencing. The Primer3 software (http://primer3.ut.ee/) was used for the primer design, according to the reference sequences in the NCBI Gene database. The primers for the *COL5A1* mutation validation were synthesized by Sangon Biotech (Shanghai, China): *COL5A1*-Forward, GACTCGGGTCTTCTGGTTC and *COL5A1*-Reverse, and TTTGGTTCAGTAGCTGGTATG. All PCR products were subjected to Sanger sequencing using an ABI3730 automated sequencer (PE Biosystems, Foster City, CA, USA) and analyzed by Lasergene SeqMan (DNASTAR, Madison, WI, USA).

### 2.5. *In Silico* Prediction Analysis

The *in silico* prediction analysis of genomic variants was performed using the Human Splicing Finder [18], SpliceMan [19], and ExonScan [20].

## 3. Results

### 3.1. Clinical Findings

**Figure 2:**
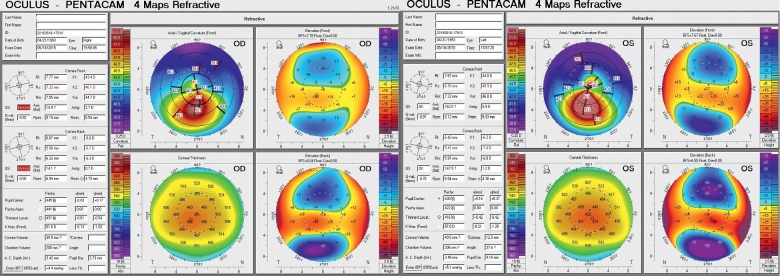
Corneal topography of the proband (Pentacam). OD = right eye; OS = left eye.

The proband (Patient III:4, Figure 1), who was a 25-year-old male, had defective vision since the age of 21 and has been previously diagnosed with KC. After the physical examination, the UDVA was 10/50 and 6/50 in the right and left eye, respectively, while the CDVA was 10/50 with −5.75DS/−3.75DC × 170 and 10/50 with −6.00DS/−5.50DC × 155 in the right and left eye, respectively. The slit-lamp examination revealed thinning and protrusion in the central cornea of all individuals with KC. In addition, significant inferior steepening plus irregular astigmatism were observed through corneal topography, indicating the onset of KC. The mean posterior elevation of 45 *μ*m and 56 *μ*m was observed in the right and left eye, respectively, for all individuals with KC. The central corneal thickness was 431 *μ*m and 416 *μ*m in the right and left eye, respectively. The anterior surface keratometry was 43.4/46.1 D and 44.0/49.9 D in the right and left eye, respectively (right eye maximum K, 51.1 D; left eye maximum K, 56.8 D; Figure 2). Patient III:3, who was a 23-year-old female diagnosed with KC at the age of 22, and all other affected family members also underwent the physical examinations, including visual acuity, slit-lamp, and corneal topography and were diagnosed with corneal ectasia. The clinical grades for these individual KC patients and the correlation with CCT are summarized in Table 1.

### 3.2. Identification of a Novel Genetic Variant

**Figure 3:**
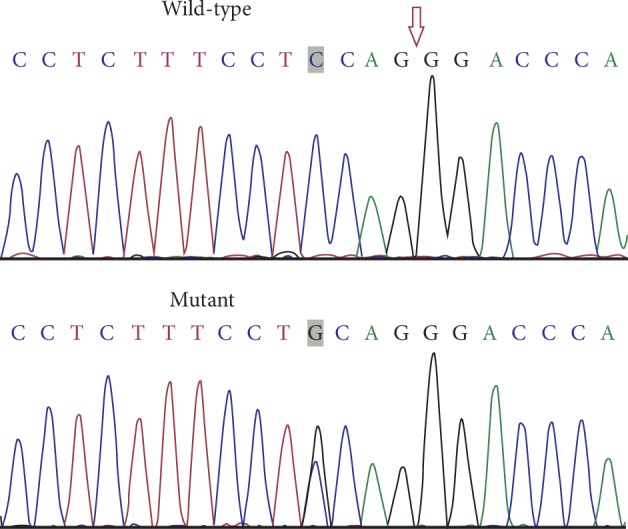
The comparison of the wild-type (III:2) and mutant (III:4) sequences revealed that the latter harbors a IVS50-4C > G splice-site mutation. The arrow indicates the exon-intron boundary.

Comparing KC patients to healthy individuals, *COL5A1* is the only non-SNP variant identified with a frequency of ≤1% within the intron, while other variants (SNPs) were identified at the exon (Table 2). The novel heterozygous genetic variant is an acceptor-splice-site (IVS50-4C > G) mutation in intron 50. This mutation was found in all affected individuals of the three-generation KC family (Figure 3). The splice-site mutation (IVS50-4C > G) in *COL5A1* was absent in both the unaffected family members and unrelated healthy controls, as well as in the 1000 Genomes Project (http://browser.1000genomes.org/index.html).

### 3.3. Effects of the Splice-Site Mutation

The *in silico* Human Splicing Finder prediction program suggests that the mutation likely has a minimal impact on splicing, while the SpliceMan predictor program suggests that this acceptor-splice-site mutation could disrupt the splicing (a prediction score of 60%).

The analysis of exon 50 and the two flanking exons (exon 49–50–51) using the ExonScan software to predict the strength of the splice-site and the presence of splicing regulatory elements predicted the alteration of the maximum entropy score of the 3' splice-site (beginning of the 50^th^ exon), which may lead to the skipping of exon 50 that was downstream of the splice-site mutation during transcription. Thus, the predicted disruption in gene splicing due to the presence of the IVS50-4C > G splice-site mutation may potentially lead to an abnormal and unstable type V collagen structure.

## 4. Discussion

Genetic inheritance, as a probable mechanism of KC pathogenesis, has been implicated in some cases of KC, and several genes that may confer the disease have been identified in recent years [4, 6]. However, the identification of more candidate KC genes, especially genes that are closely correlated to CCT, such as those of the connective tissue or extracellular matrix, is imperative to fully understand KC pathogenesis. Next-generation sequencing (NGS) and WES technology are powerful tools for screening families who particularly carry inherent KC. KC affects vision and significantly reduces the quality of life of affected individuals. Hence, the early diagnosis and intervention of KC can significantly boost clinical treatment and delay KC progression [21]. High-frequency gene variants could also serve as a useful diagnostic/prognostic marker for disease progression.

The corneal stroma is enriched with collagen fibrils. The genomic loci associated with CCT contain genes, such as *COL1A1*, *COL1A2*, and *COL5A1*, which code for various collagen proteins [4, 7]. The *COL5A1* gene, which encodes type V collagen alpha chain subunit 1, has relatively low abundance, but is widely distributed in the body, including the blood vessel walls, fetal membranes, skin, and cornea [22]. Type V collagen plays a critical role in organizing heterotypic type I/V collagen fibrils [22, 23]. In the cornea, type V collagen has been found to coaggregate with type I collagen to assemble into heterotypic fibrils, thereby regulating the diameter of heterotypic fibers formed by type I and V collagen.

The present study is the first to identify a rare acceptor-splice-site mutation IVS50-4C > G in the *COL5A1* gene, which was only detected in affected individuals of the three-generation family. This splice-site mutation was absent in unaffected family members, population-matched controls, and the 1000 Genomes Project. The acceptor-splice-site mutation is located in intron 50, which is upstream of exon 50. However, the *in silico* analysis to assess the potential impact of the splice-site mutation on the KC phenotype using the Human Splicing Finder program and SpliceMan predictor program presented contradicting results. Hence, future studies are necessary to clarify the impact of the novel splice-site mutation on the type V collagen structure.

The SpliceMan predictor program predicted the positive association between the novel splice-site mutation and KC pathogenesis. Indeed, since most type V collagen defects produce a nonfunctional *COL5A1* allele [16], the investigators deduced that the novel splice-site mutation identified in the present study may contribute to KC pathogenesis. Merely few variants have been reported to affect the structure of type V collagen, such as splicing errors in the collagen triple helix and C-propeptide mutations [24], thereby suggesting that the novel splice-site mutation identified may cause structural changes in the collagen protein and affect the assembly of the collagen triple helix. The present analysis results obtained from the ExonScan software support the potential disease relevance of the IVS50-4C > G splice-site mutation.

## 5. Conclusion

In summary, a rare acceptor-splice-site mutation IVS50-4C > G in *COL5A1* was found in all individuals with KC in a three-generation Indian family. The *in silico* analysis of the impact of splicing and its regulatory elements suggest that this mutation could disrupt *COL5A1* splicing and affect the resulting type V collagen protein structure and stability. The findings of the present study serve as a starting point for the future research dissection of the effect of this mutation on type V collagen function and structure and KC pathogenesis.

## Figures and Tables

**Table 1:** Clinical grades of KC members and changes in corneal thickness.

KC member	CCT (*μ*m)	Clinical grade
I1	378 (OD), 407 (OS)	2
II2	409 (OD), 414 (OS)	2
II3	396 (OD), 426 (OS)	2
II7	438 (OD), 420 (OS)	2
III1	410 (OD), 425 (OS)	2
III3	436 (OD), 429 (OS)	2
III4	431 (OD), 416 (OS)	2
III5	448 (OD), 423 (OS)	2
III6	453 (OD), 458 (OS)	2
III8	440 (OD), 447 (OS)	2

OD: oculus dextrus (left eye); OS: oculus sinister (right eye).

**Table 2:** Preliminary KC candidate variants.

Gene	Phenotype	Variation
*COL5A1*	Heterozygote, splice site	IVS50-4C > G
*VSX1*	Heterozygote, synonymous	rs10943299
TGFBI	Heterozygote, synonymous	rs1054124
TGFBI	Heterozygote, synonymous	rs1133170
TGFBI	Heterozygote, synonymous	rs4669
ZNF469	Heterozygote, synonymous	rs3812953
ZNF469	Heterozygote, synonymous	rs4782301
ZNF469	Heterozygote, synonymous	rs12918876

## Data Availability

The datasets generated and analyzed in the present study are available from the corresponding author upon reasonable request.
